# Neuropeptides: Developmental Signals in Placode Progenitor Formation

**DOI:** 10.1016/j.devcel.2013.07.001

**Published:** 2013-07-29

**Authors:** Laura Lleras-Forero, Monica Tambalo, Nicolas Christophorou, David Chambers, Corinne Houart, Andrea Streit

**Affiliations:** 1 Department of Craniofacial Development and Stem Cell Biology, King’s College London, Guy’s Tower Wing, Floor 27, London SE1 9RT, UK; 2 MRC Centre for Developmental Neurobiology, King’s College London, New Hunts House, London SE1 1UL, UK

## Abstract

Few families of signaling factors have been implicated in the control of development. Here, we identify the neuropeptides nociceptin and somatostatin, a neurotransmitter and neuroendocrine hormone, as a class of developmental signals in both chick and zebrafish. We show that signals from the anterior mesendoderm are required for the formation of anterior placode progenitors, with one of the signals being somatostatin. Somatostatin controls ectodermal expression of *nociceptin*, and both peptides regulate *Pax6* in lens and olfactory progenitors. Consequently, loss of somatostatin and nociceptin signaling leads to severe reduction of lens formation. Our findings not only uncover these neuropeptides as developmental signals but also identify a long-sought-after mechanism that initiates *Pax6* in placode progenitors and may explain the ancient evolutionary origin of neuropeptides, predating a complex nervous system.

## Highlights

• Anterior mesendoderm promotes sensory progenitor specification
• Neuropeptides promote lens and olfactory epithelium fate
• Neuropeptides induce Pax6, the master regulator of eye formation

## Teaser

Placode progenitors generate major components of sense organs. Lleras-Forero et al. show that neuropeptides control early placode development: mesendoderm-derived somatostatin promotes ectodermal *nociceptin* expression. Together, both peptides regulate *Pax6* in lens and olfactory progenitors. Consequently, loss of somatostatin and nociceptin signaling leads to severe reduction of lens formation.

## Introduction

In the vertebrate head, multipotent progenitor cells give rise to crucial components of sense organs and sensory ganglia (Schlosser, 2010, Streit, 2007). They reside in the ectoderm next to the anterior neural plate, where they are induced by FGFs combined with Wnt and BMP attenuation (Ahrens and Schlosser, 2005, Brugmann et al., 2004, Litsiou et al., 2005). Although they generate cells as diverse as lens fibers and olfactory sensory neurons, they are initially competent to form any placode and share a common developmental program: at neurula stages, all sensory progenitors are specified as lens and diversify later under the influence of inductive signals from surrounding tissues (Bailey et al., 2006, Baker et al., 1999, Bhattacharyya and Bronner-Fraser, 2008, Gallagher et al., 1996, Groves and Bronner-Fraser, 2000, Henry and Grainger, 1987, Jacobson, 1963, Martin and Groves, 2006, Schlosser, 2010, Streit, 2007).

One of the key factors initiating the lens program is the paired box transcription factor Pax6 (Cvekl and Duncan, 2007, Lang, 2004). Consistent with its expression in lens and olfactory precursors (anterior placode progenitors [aPPs]), Pax6 mutations in humans and mice lead to severe eye and olfactory abnormalities (Grindley et al., 1995, van Heyningen and Williamson, 2002). Indeed, Pax6 is one of the most striking examples of a master regulator: its misexpression not only induces ectopic eyes in flies and vertebrates, but its function is so conserved that vertebrate Pax6 does so in *Drosophila* (Chow et al., 1999, Halder et al., 1995). It is therefore surprising that the tissues and signals that initiate *Pax6* expression—and thus aPP specification—are unknown. Here, we demonstrate that the anterior mesendoderm is required for aPP formation and regulates *Pax6*. It does so by secreting the neuropeptide somatostatin (SST), which in turn activates *nociceptin* (*Noc*) in the overlying ectoderm. Together, these neuropeptides control aPP fate as an early step in lens and olfactory epithelium development.

## Results

### Identification of Somatostatin and Nociceptin as Potential Developmental Signals

Consistent with its function, *Pax6* is initially confined to future lens and olfactory cells (aPPs). However, chick posterior placode progenitors (pPPs; inner ear and epibranchial ganglia) upregulate *Pax6* within only 5 hr when cultured in isolation and ultimately turn into lenses (Bailey et al., 2006), providing an experimental paradigm to screen for new *Pax6* regulators. Transcriptome comparison of four different cell populations (HH6 aPPs and pPPs before and after 5 hr culture) reveals 136 *Pax6* coregulated transcripts. Among these, only four encode signaling molecules, including the propeptide for the opioid-related *Noc* (Figure S1 available online); the receptor for another neuropeptide, SST, is enriched in aPPs. This raises the intriguing possibility that in addition to their well-known functions in the adult nervous system and neuroendocrine modulation (Gahete et al., 2010), they may also play a role during development.

**Figure 1:**
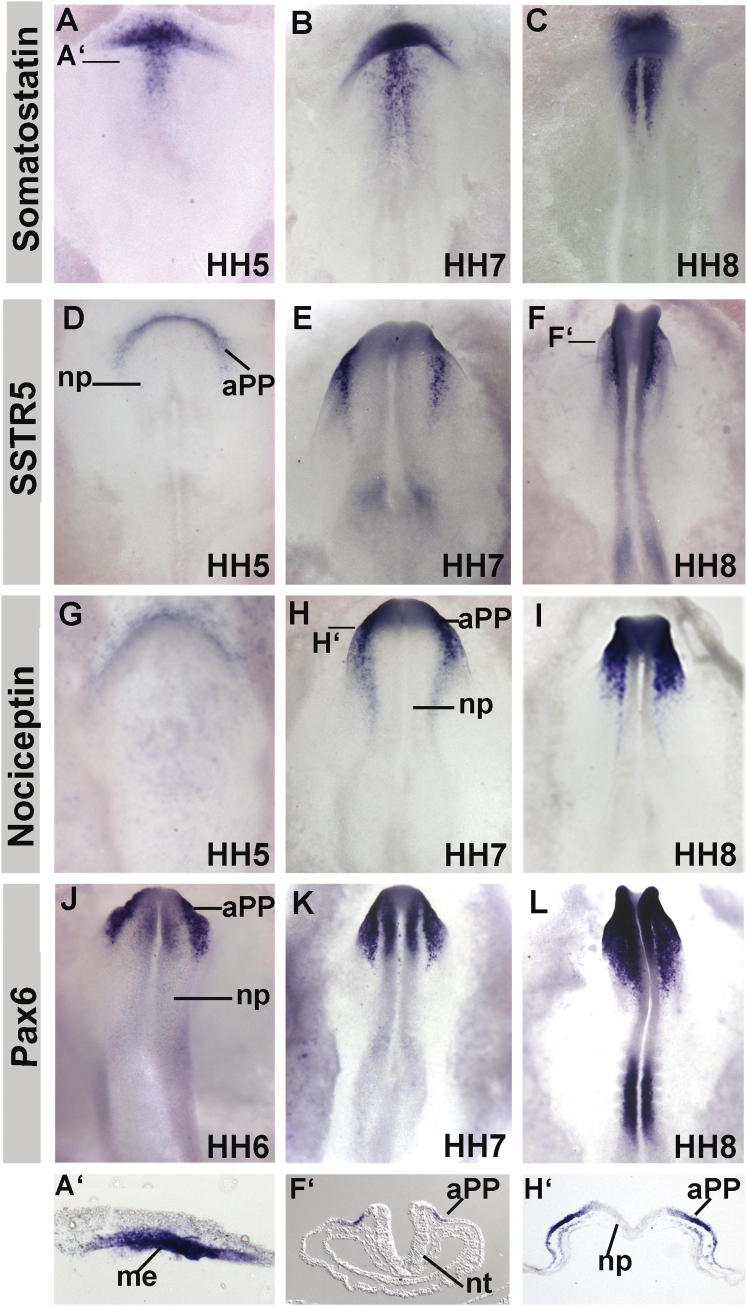
Expression of *SST*, *SSTR5*, *Noc*, and *Pax6* In situ hybridization for *SST* (A–C ventral view; A′ transverse section), *SSTR5* (D–F dorsal view; F′ transverse section), *Noc* (G–I dorsal view; I′ transverse section), and *Pax6* (J–L dorsal view). Stages are indicated in the right corner of each panel. Black lines in (A)–(L) indicate the level of the sections. aPP, anterior placode progenitors; me, mesendoderm; np, neural plate; nt: neural tube. See also Figure S1.

We therefore surveyed the expression of *Noc* and *SST* and their receptors from primitive streak to early somite stages. The *SST* prepropeptide and the processed peptide are expressed in the anterior mesendoderm underlying *Pax6*^+^ aPPs (Figures 1A–1C, 1A′, S1Ca–S1Cf, S2A, and S2B). In contrast, its receptor *SSTR5* and *Noc* (Figures 1D–1I, 1F′, and 1H′) are restricted to aPPs in the overlying ectoderm, where they colocalize with *Pax6* (Figures 1J–1L); both are downregulated rapidly in aPPs after the HH8. Thus, *Noc* and *SSTR5* represent aPP-specific transcripts. Like *Noc* mRNA, the processed peptide is present in the ectoderm (Figures S1Cg–S1Cl). Noc signaling is mediated by its cognate G protein-coupled receptor opiate receptor-like 1 (OPRL1) and, due to a change in the N-terminal amino acid in most nonmammalian vertebrates, by δ, κ, and μ opioid receptors (Danielson et al., 2001) (Figures S1Aa and S1Ab). At neural plate stages, all opioid and other SST (*STTR1*, *STTR3*, *STTR4*) receptors are broadly expressed in the ectoderm including placode progenitors (Figures S2Ac–S2Aj and S3Aa–S3Al). Thus, the expression of both neuropeptides and their receptors is consistent with a role in initiating *Pax6* expression and in specifying progenitors for the olfactory epithelium and the crystalline lens.

### Somatostatin Signaling from the Anterior Mesendoderm Promotes Placode Progenitor Fate

**Figure 2:**
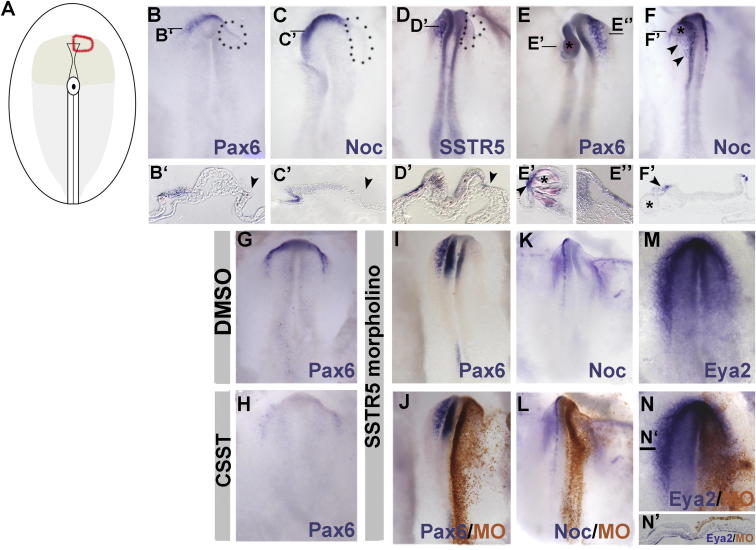
SST Is Required for aPP Character (A) Unilateral ablation of axial and paraxial mesendoderm (red line); light gray indicates mid- and hindgut endoderm, and gray shows foregut endoderm. (B–F) *Pax6* (B and B′), *Noc* (C and C′), and *SSTR5* (D and D′) after mesendoderm ablation (dotted lines); *Pax6* (E and E′) and *Noc* (F and F′) after mesoderm ablation and SST bead graft. SST-coated beads (^∗^) restore *Pax6* (E–E″) and *Noc* (F and F′) after ablation (arrowheads). Lines in (B)–(F) indicate the level of sections shown in (A′)–(E′). (G and H) *Pax6* after DMSO (G) or CSST (H) treatment. (I–N) *Pax6* (I and J), *Noc* (K and L), and *Eya2* (M, N, and N′) in SSTR5 morphants. (J), (L), and (N) show the same embryos as in (I), (K), and (M), respectively, after MO detection (brown). See also Figure S2.

*SST* is expressed in the anterior mesendoderm, a tissue implicated in forebrain patterning (Dale et al., 1997, Foley et al., 1997, Wilson and Houart, 2004, Withington et al., 2001). To test whether mesendoderm-derived signals are required for aPP identity, we ablated this tissue unilaterally in HH4^+^/HH5^−^ embryos (Figures 2A, S2Ba, and S2Bb). We find that *Pax6* (1 out of 14, 7% *Pax6*^+^; Figures 2B and 2B′) and the general PP marker *Eya2* (0 out of 4 *Eya2*^+^; Figure S2Be) are absent 5–6 hr thereafter, whereas sham-operated embryos are normal (Figure S2Bf). Likewise, *Noc* transcripts are reduced at 5–6 hr and completely lost 16 hr after ablation (0 out of 7 *Noc*^+^; Figures 2C and 2C′), as is *SSTR5* (0 out of 9 *SSTR*^+^; Figures 2D and 2D′). In contrast, the posterior ectoderm marker *Gbx2* (n = 4) and nonneural ectoderm marker *Dlx5* (n = 4) are unaffected (Figures S2C and S2D). Can SST rescue the expression of aPP markers? After mesendoderm removal, local exposure to SST-, but not DMSO-, coated beads (Figures S2Bg–S2Bh′) rescues *Pax6* (3 out of 3 *Pax6*^+^; Figures 2E–2E″) and *Noc* expression (5 out of 5 *Noc*^+^; Figures 2F and 2F′) after 16 hr culture. Thus, the anterior mesendoderm provides key signals to promote aPP identity, SST being one of these signals.

To assess whether SST signaling is required for aPP character, we used two different approaches. First, HH4 embryos were cultured with the SST antagonist cyclosomatostatin (CSST) or vehicle control (DMSO). At HH6/HH7, *Pax6* expression is absent in CSST (4 out of 18, 22% *Pax6*^+^; Figure 2H) but present in DMSO-treated controls (6 out of 6 *Pax6*^+^; Figure 2G). Second, we asked whether the receptor SSTR5 mediates SST function. Control or SSTR5 translation-blocking morpholinos (MOs) were electroporated into future aPPs at HH4. Like mesendoderm ablation, SSTR5 knockdown leads to a loss of *Pax6* (1 out of 13, 8% *Pax6*^+^; Figures 2I and 2J), *Noc* (5 out of 19, 26% *Noc*^+^; Figures 2K and 2L), and *Eya2* (0 out of 6 *Eya2*^+^; Figures 2M, 2N, and 2N′) at HH6-8, whereas control MOs have no effect (Figures S2Bi–S2Bn). Together, these results show that SST signaling from the anterior mesendoderm is crucial for the specification of lens and olfactory progenitors by controlling the onset of *Pax6* and other PP-specific transcripts.

### Signals from the Posterior Head Mesoderm Repress aPP Markers

**Figure 3:**
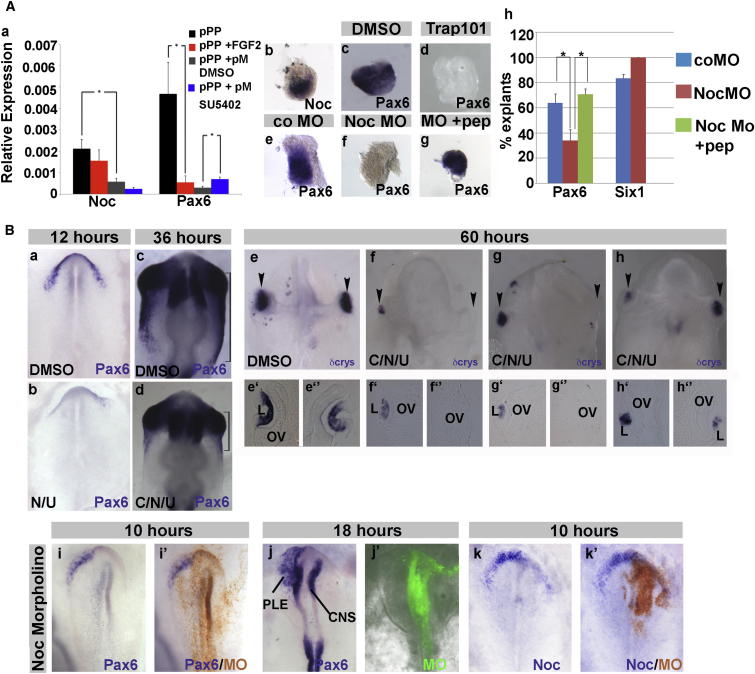
Noc Is Required for aPP Character (A) Regulation of aPP markers in vitro. (a) Quantification of *Noc* and *Pax6* expression by NanoString nCounter in pPP explants cultured alone (black), with FGF2 (red), with posterior head mesoderm (pM; gray), or with posterior head mesoderm and SU5402 (blue). Bars represent means of normalized values ± SE. The asterisk (^∗^) indicates significant differences: p < 0.05. (b) Cultured pPP explants initiate *Noc*. (c and d) *Pax6* after DMSO (c) or TRAP101 (d) treatment. (e–g) *Pax6* in explants cultured with control MOs (e), Noc splice-blocking MOs (f), or Noc MOs and peptide (g). (h) Graph showing Noc knockdown effect on *Pax6* and *Six1*. Bars represent mean values ± SE. The asterisk (^∗^) indicates significant differences: p < 0.05. See also Figure S3. (B) Noc is required for aPP fate in vivo. (a and b) HH4^+^/HH5^−^ embryos cultured for 12 hr in DMSO (a) or opioid receptor inhibitors (b; N/U: naloxone, UFP101). (c and d) *Pax6* in HH4^+^/HH5^−^ embryos treated with DMSO (c) or CSST (C) for 36 hr, naloxone (N) and UFP101 (U); compare brackets in (c) and (d). (e–h) *δ-crystallin* expression in embryos cultured for 60 hr from HH4^+^/HH5^−^ with DMSO (e) or CSST (C), naloxone, and UFP101 (f–h). (f)–(h) show the range of phenotypes, and (e′)–(h″) show sections through the left and right lens (L) regions of the same embryos shown in (e)–(h). OV, optic vesicle. (i–k′) After electroporation of Noc ATG MOs at HH4^−^, embryos were cultured for 10 hr (i and i′, and k and k′) or 18 hr (j and j′). Brown (i′ and k′) and green (j′) indicate electroporated cells.

Although *Pax6* and *Noc* are upregulated in explanted pPPs (Figures S1 and 3A), they are restricted to the aPP region in the embryo (Figure 1), suggesting that anterior fates are actively repressed in vivo. A possible source for such repressive signals is the mesoderm underlying pPPs. To test this, we analyzed *Pax6* and *Noc* expression in pPP explants cultured with and without posterior mesoderm (pM). Indeed, we find that both transcripts are repressed by mesoderm-derived signals (Figure 3Aa). pPPs normally give rise to otic and epibranchial placodes with FGFs being potent inducers of a common otic-epibranchial progenitor domain (Freter et al., 2008, Groves and Bronner-Fraser, 2000, Martin and Groves, 2006, Yang et al., 2013). To test whether FGF signaling is sufficient for aPP inhibition, we cultured pPP explants with FGF2; this induces the otic-epibranchial marker Pax2 (data not shown) (Freter et al., 2008, Groves and Bronner-Fraser, 2000, Martin and Groves, 2006, Yang et al., 2013). Although *Noc* expression does not change significantly when compared to controls, *Pax6* is significantly repressed (Figure 3Aa). To test whether mesoderm-derived FGF signaling is required for *Pax6* repression, we treated pPP/pM explants with the FGF antagonist SU5402; this rescues *Pax6* expression partially but does not restore the expression levels observed in the absence of mesoderm (Figure 3Aa). Together, these findings show that posterior head mesoderm plays a role in patterning the placodal domain by simultaneously promoting posterior and repressing anterior character. Although FGF signaling is involved in this process, other unidentified pathways must cooperate.

### Nociceptin Promotes aPPs

*Noc* was identified as a *Pax6* coregulated gene (Figure S1); both genes are coexpressed in aPPs (Figure 1), and like *Pax6*, *Noc* is rapidly upregulated in explanted pPPs (n = 19 out of 22; 86% *Noc*^+^; Figure 3Ab). To test whether *Pax6* upregulation depends on Noc signaling, we used an OPRL1 antagonist. *Pax6* transcripts are present in DMSO-treated control pPP explants (17 out of 23, 73% *Pax6*^*+*^; Figure 3Ac) but absent when Noc signaling is inhibited (11 out of 31, 35% *Pax6*^+^; Figure 3Ad). Likewise, Noc splice MOs, but not control MOs, prevent *Pax6* initiation (Figures 3Ae, 3Af, and 3Ah; controls, 39 out of 64, 61% *Pax6*^+^; experimental, 27 out of 73, 34% *Pax6*^+^). This effect is rescued by the addition of Noc peptide (13 out of 17, 76% *Pax6*^+^; Figures 3Ag, 3Ah, and S3Ca), demonstrating the specificity of the MOs. In contrast, the generic PP marker *Six1* does not change after Noc knockdown (Figure 3Ah, 23 out of 23 *Six1*^*+*^; Figure S3Ca), indicating that Noc specifically promotes anterior character.

To confirm that Noc is required for aPP specification in vivo, we used two strategies. First, HH4 chick embryos were treated with OPRL1 and opiate receptor antagonists; this leads to loss of *Pax6* at HH6 (Figure 3Bb; 0 out of 8 *Pax6*^+^), unlike DMSO-treated controls (Figure 3Ba; 8 out of 11, 72% *Pax6*^+^). Second, we electroporated translation- or splice-blocking Noc MOs, alone or in combination, into stage HH4 chick embryos, targeting future lens and olfactory cells. These produce identical phenotypes: at early somite stages, the expression of general placode progenitor markers (Figures S3Be–S3Bh′; *Six1*^+^, n = 8; *Eya2*^+^, n = 7), the neural crest marker *Pax7* (Figures S3Ba, S3Bb, and S3Bb’; n = 5), and *Otx2* (Figures S3Bc, S3Bd, and S3Bd′; n = 7), an anterior ectoderm marker expressed prior to *Pax6* and *Noc*, are all unaffected. However, like in vitro, in Noc morphants, *Pax6* is severely reduced in lens and olfactory progenitors at head process and early somite stages (Figures 3Bi–3Bj′, 4 out of 14, 29% *Pax6*^+^; Figure S3Cb) as is *Noc* itself (0 out of 4 *Noc*^+^; Figures 3Bj and 3Bj′), whereas control MOs have little effect (Figures S2I–S2L; 7 out of 10, 70% *Pax6*^+^; 7 out of 11, 64% *Noc*^+^). In contrast, the CNS domain of *Pax6* is unaffected as is the expression of *Six3* (n = 9; data not shown) and *Ganf* (n = 2; data not shown) in the forebrain. Thus, Noc does not influence neural plate formation but is required to regulate anterior placode fates by controlling its own expression and that of the master regulator *Pax6*.

So far, our results show that mesendoderm-derived SST initiates *Noc* expression in the overlying ectoderm and that both signals are required for aPP-specific gene expression. Do SST and Noc act in a linear pathway? If so, nociceptin should rescue the SST phenotype. We therefore ablated anterior mesendoderm (the source of SST) or knocked down SSTR5 followed by a graft of Noc-coated beads. Activation of Noc signaling does not rescue *Pax6* expression in the absence of SST (Figure S3D; n = 13). Thus, the two neuropeptides act in parallel, and both are required for *Pax6* expression and, consequently, for aPP specification.

### Lens Defects in the Absence of SST and Noc Signaling

*SST* and *Noc* are transiently expressed in the anterior mesendoderm and anterior preplacodal ectoderm, respectively, and participate in aPP specification by regulating *Pax6* (see above). Does the loss of aPP character affect placode formation at later stages? To assess this, we treated embryos with inhibitors of Noc and SST signaling starting at HH4/HH5^−^. This reduces *Pax6* in the surface ectoderm, but not in the brain, at HH10/HH11 (Figures 3Bc and 3Bd; 7 out of 14, 50% Pax6 reduction; controls: 13 out of 14, 93% normal). At embryonic day 3 (E3), phenotypes vary slightly, with only 12.4% (n = 24) showing normal expression of the lens differentiation marker *δ-crystallin* compared to 70% of DMSO-treated controls (n = 20). Lens placodes or vesicles are absent uni- (12.4%) or bilaterally (16.6%) or are substantially smaller (58.3%; Figures 3Bf–3Bh and 3Bf′–3Bh″) than in controls (Figures 3Be and 3Be′; n = 20; 30% with unilateral small placode). Although patches of *δ-crystallin* show typical placode morphology, they remain small and never invaginate to form a vesicle. In severe cases, optic vesicle morphology is affected because lens-derived signals are required for its normal development (Coulombre and Coulombre, 1964, Yamamoto and Jeffery, 2000, Chow and Lang, 2001). Thus, SST and Noc signaling are critical for aPP specification and therefore for normal lens formation.

### Somatostatin and Nociceptin Functions Are Conserved in Zebrafish

**Figure 4:**
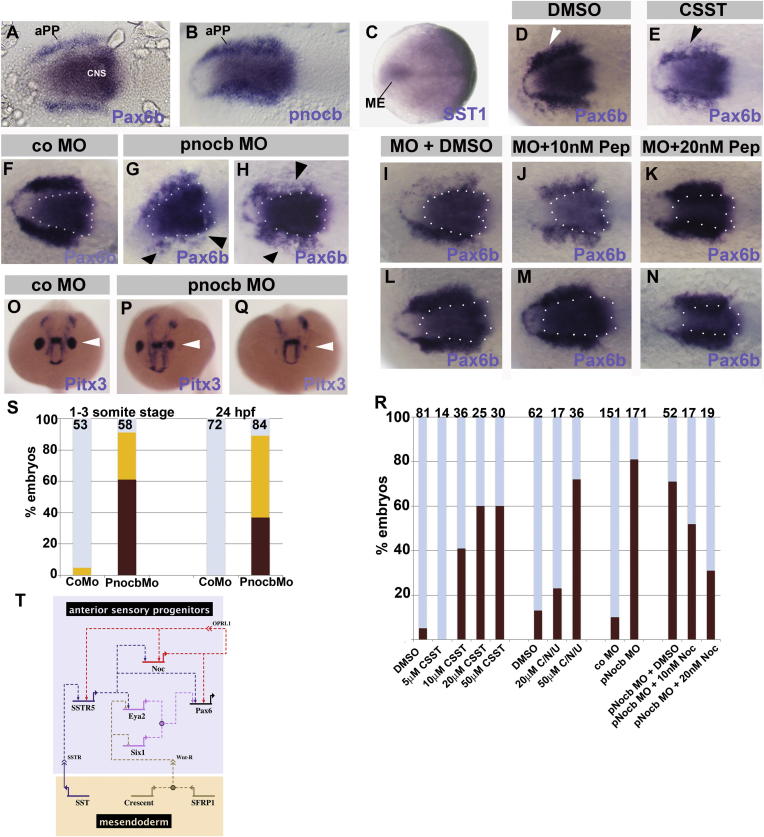
SST and Noc Control aPPs in Zebrafish (A–C) Expression of *Pax6b* (A), *pnocb* (B), and *SST1* (C) in zebrafish at early somite stages: dorsal views, anterior to the left. (D and E) *Pax6b* reduction by CSST (E; arrowhead), but not by DMSO (D; white arrowhead). (F–H) Embryos were injected with control (F) or pnocb MOs (G and H); the latter show *Pax6b* reduction (arrowheads in G and H). Dotted lines indicate CNS expression of *Pax6b*. (I–N) pnocb ATG (I–K) or control (L–N) MO-injected embryos were incubated in DMSO (I and L) or Noc peptide (J, K, M, and N). Dotted lines indicate CNS *Pax6b* expression. (O–Q) Embryos were injected with control (O) or pnocb MOs (P and Q). At 24 hpf (frontal views), *Pitx3* expression reveals asymmetric, small (P; arrowhead) or almost absent lenses (Q; arrowhead). (R) Graph shows *Pax6b* reduction after SST inhibition, SST and Noc inhibition, in Noc morphants and Noc morphants + Noc peptide. Numbers for each treatment are at the top. Brown bars indicate embryos with phenotype, blue bars show normal embryos. (S) Quantification of pnocb morphants with *Pitx3* reduction (yellow) or loss (brown) at early somite stages or 24 hpf. (T) Model for neuropeptide function.

To assess whether neuropeptide function in anterior placode precursor formation is conserved across species, we turned to zebrafish and cloned prepronociceptin b (pnocb). As in chick, *pnocb* is coexpressed with *Pax6b* in aPPs (Figures 4A and 4B), whereas *SST1* (Devos et al., 2002) is expressed in the mesendoderm (Figure 4C). Like in chick, inhibition of SST signaling using the antagonist CSST leads to disruption of *Pax6b* at neural plate stages in a dose-dependent manner (Figures 4D, 4E, and 4O). Noc knockdown using two different MOs alone or in combination results in uni- or bilateral reduction of *Pax6b* (Figures 4G, 4H, and 4O; 32 out of 171, 19% normal *Pax6b*) and *Pitx3* (Figure 4P; 5 out of 58, 9% *Pitx3*^*+*^) in aPPs at neural plate stages. *Pax6b* expression is rescued by growing morphants in the presence of Noc peptide (Figures 4I–4K and 4O). In *pnocb* morphants, the general PP markers *Eya1* and *Six1* are unaffected (data not shown), suggesting that, like in chick, aPPs retain placode progenitor identity but lose their anterior character. In contrast to *Pax6b* reduction in aPPs, its expression in the neural plate is unaffected, as are the neural markers *Rx3*, *Emx1*, *Six3*, and *Irxb* until at least the ten-somite stage (data not shown; n > 25 for each marker). These results show that nociceptin signaling primarily affects placode, but not neural plate, development.

Consistent with the loss of early *Pax6b*, Noc morphants show variable eye phenotypes after 24 hr: their lenses are smaller, asymmetric, or absent (n = 84; Figures 4L–4N and 4P), and as a consequence, the optic vesicles are reduced in size. Simultaneous inhibition of SST and Noc phenocopies the loss of each pathway individually (n = 54; data not shown). Thus, like in chick, *Noc* is coexpressed with *Pax6b* in placode progenitors at the border of the anterior neural plate, whereas *SST1* is expressed in the underlying mesendoderm. Both contribute to the specification of aPPs, and their loss leads to abnormal eye development in chick and zebrafish.

## Discussion

Our findings reveal a function for the anterior mesendoderm in controlling aPP fates as an early step for lens and olfactory development. We identify two neuropeptides, SST and Noc, mediating this process in amniotes and anamniotes (Figure 4T). Mesendoderm-derived SST promotes aPP identity in the overlying ectoderm by regulating *Eya2* and *Noc*. In turn, Noc controls its own expression and, together with SST, the onset of *Pax6*, a key regulator of eye and olfactory fates.

Mice lacking Noc, SST, and their receptors have been generated (Köster et al., 1999, Low et al., 2001, Nishi et al., 1997, Zeyda et al., 2001, Zeyda and Hochgeschwender, 2008). Adult animals do not display obvious sense organ phenotypes, for which several other explanations are possible. First, the mutants have not been examined for defects in lens and olfactory progenitors—the effects of the mutants may be transient or subtle. With respect to SST, a robust phenotype would only be expected when all four SSTRs expressed in sensory progenitors are ablated. Furthermore, a second peptide, cortistatin, is often coexpressed with SST, signals through all SSTRs, and may thus compensate for the absence of SST (Gahete et al., 2008, Zeyda and Hochgeschwender, 2008). Finally, the mammalian Noc prepropeptide contains a second peptide, nocistatin, a Noc antagonist, which is absent in nonmammalian vertebrates (Danielson et al., 2001, Okuda-Ashitaka et al., 1998). This may account for subtle or lack of phenotype in mice. Here, using different tools including tissue ablation, morpholinos, and drugs to interfere with SST and Noc function, we reveal their role in aPP specification. Whether neuropeptides have a similar function in rodents and other mammals remains to be discovered; however, our results are consistent in both amniotes and anamniotes.

Once placode progenitors are specified, they have an autonomous tendency to form a lens when cultured in isolation, regardless of their later fate (Bailey et al., 2006). They do so by initiating *Pax6* followed by the transcription factors controlling lens-specific *δ-crystallin*. However, in the embryo, pPPs never express *Pax6* and never contribute to the lens, suggesting that, in these cells, aPP character must be actively repressed. Here, we show that this is indeed the case: signals from the posterior head mesoderm inhibit aPP-specific genes partly through FGF (this study). When grafted anteriorly, the same mesoderm induces posterior character in the adjacent ectoderm (Kil et al., 2005). In contrast, anterior mesendoderm promotes anterior placode identity, and we show that SST signaling participates in this process by initiating *Noc* and *Pax6* expression. Thus, signals from the underlying mesoderm play a crucial role in patterning the placode progenitor field along the rostro-caudal axis.

In agreement with their role in aPP specification, SST, Noc, and their respective receptors are expressed prior to *Pax6*, and their loss leads to severe reduction of the lens. However, they are likely to act in concert with other signals to impart anterior identity: Wnt and BMP attenuation is important for placode progenitor induction (Ahrens and Schlosser, 2005, Brugmann et al., 2004, Litsiou et al., 2005), whereas retinoic acid, BMP7, and FGF-like signals have been implicated in forebrain patterning (Dale et al., 1997, Halilagic et al., 2003, Sanchez-Arrones et al., 2012). Our data show that SST and Noc specifically control placodal fate, without affecting neural plate patterning. Together, these observations suggest that spatial and temporal integration of different pathways is crucial in aPP specification and in establishing the differences between neural and nonneural components of the cranial sensory nervous system.

Neuropeptides and neurotransmitters are ancient signaling molecules already present in primitive deuterostomes and other invertebrates (Buznikov et al., 2005, Buznikov et al., 2010, Coates et al., 2000, Conzelmann et al., 2011, Elphick, 2010, Elphick and Thorndyke, 2005, Hauser et al., 2006, Hauser et al., 2008). They predate the origin of the CNS. In these systems, they control proliferation and growth (Lauder, 1993); neurotransmitters like serotonin control oocyte maturation and cleavage as well as neuronal differentiation and function (Buznikov et al., 2005, Buznikov et al., 2010). In vertebrates, nonneuronal functions of serotonin include craniofacial development and left-right asymmetry (Levin et al., 2006, Reisoli et al., 2010). The opioid system appeared about 450 million years ago with the emergence of gnathostomes (Larhammar et al., 2009), whereas other neuropeptides were already present earlier (Coates et al., 2000, Elphick, 2010, Elphick and Thorndyke, 2005, Hauser et al., 2006, Hauser et al., 2008). Our findings define unexpected functions for SST and Noc signaling in vertebrate embryos outside the CNS, where they influence cell fate and tissue morphogenesis, suggesting that these may be the ancestral roles of these small molecules, whose functions in the nervous system were co-opted much later during evolution.

## Experimental Procedures

### Chick Embryo Experiments

All chick experiments involve embryos younger than E10 and do not require a UK Home Office license. Embryos were cultured in New (Stern and Ireland, 1981) or Cornish (Nagai et al., 2011) pastry culture. Mesendoderm ablations were performed at HH5^−^. Beads coated with SST, nociceptin, or DMSO (control) were grafted into the ablated area.

CSST (cyclo(7-aminoheptanoyl-Phe-D-Trp-Lys-Thr[Bzl]); Sigma-Aldrich) and naloxone (Sigma-Aldrich) were prepared in DMSO (1 mM), UFP101 (Sigma-Aldrich) in H_2_O (1 mM). For inhibition studies, embryos were preincubated for 1 hr in SST and/or Noc antagonists (1 μM each) or DMSO (0.1%; control) and cultured in their presence for 5–60 hr. For knockdown, experiments were performed using MOs (Gene Tools), which were electroporated as described by Voiculescu et al. (2008).

HH5^+^-6 pPP explants were cultured as described by Bailey et al. (2006). Collagen gels and culture media were supplemented as required with nociceptin antagonists, nociceptin, MOs, FGF2, or SU5402. For coculture with posterior head mesoderm, pPP and mesoderm were dissected separately and recombined before culture.

Embryos and explants were processed for in situ hybridization (Streit et al., 1998) using DIG-labeled antisense probes. For histological sections, embryos were embedded in paraffin and sectioned transversally at 15 μm.

### NanoString nCounter

For each experimental condition, eight to ten pPP explants were lysed in lysis buffer (Ambion). Total RNA was hybridized with capture and reporter probes according to the nCounter Gene Expression Assay Manual. Following washing, target/probe complexes were immobilized for data collection in the nCounter Digital Analyzer. Each experiment was repeated three times on independent occasions. Mean value ± SD for Pax6 and Noc was extracted from the normalized data.

### Cloning of Zebrafish pnocb and Functional Experiments

All zebrafish studies were performed with approval from the UK Home Office under a HO project license to C.H. Zebrafish pnocb was cloned by RT-PCR from 48 hpf embryo cDNA. pnoc or control MOs were injected at the one- to four-cell stage at a concentration of 1.8 ng/embryo. Embryos were grown at 28°C until the desired stage. For inhibition and rescue experiments, dechorionated embryos were incubated in appropriate compounds from 4 to 5 hpf until they had reached the desired stage.

## Accession Numbers

The Gene Expression Omnibus accession number for the microarray data reported in this paper is GSE48116.

## Supplemental Information

Document S1. Supplemental Experimental Procedures and Figures S1–S3
