# Supplementary material for: Neuropeptides: Developmental Signals in Placode Progenitor Formation
Source: Dev Cell. 2013 Jul 29;26(2):195–203. doi: 10.1016/j.devcel.2013.07.001 (PMC3748341; doi:10.1016/j.devcel.2013.07.001)
Supplement: Document S1. Supplemental Experimental Procedures and Figures S1–S3 [file mmc1.pdf]

Developmental Cell, Volume *26*

Supplemental Information

Neuropeptides: Developmental Signals

in Placode Progenitor Formation

Laura Lleras-Forero, Monica Tambalo, Nicolas Christophorou, David Chambers, Corinne Houart, and Andrea Streit

Inventory of supplementary material

Supplementary Figure 1: relates to Figure 1

Supplementary Figure 2: relates to Figure 2

Supplementary Figure 3: relates to Figure 3

Supplementary Experimental Procedures

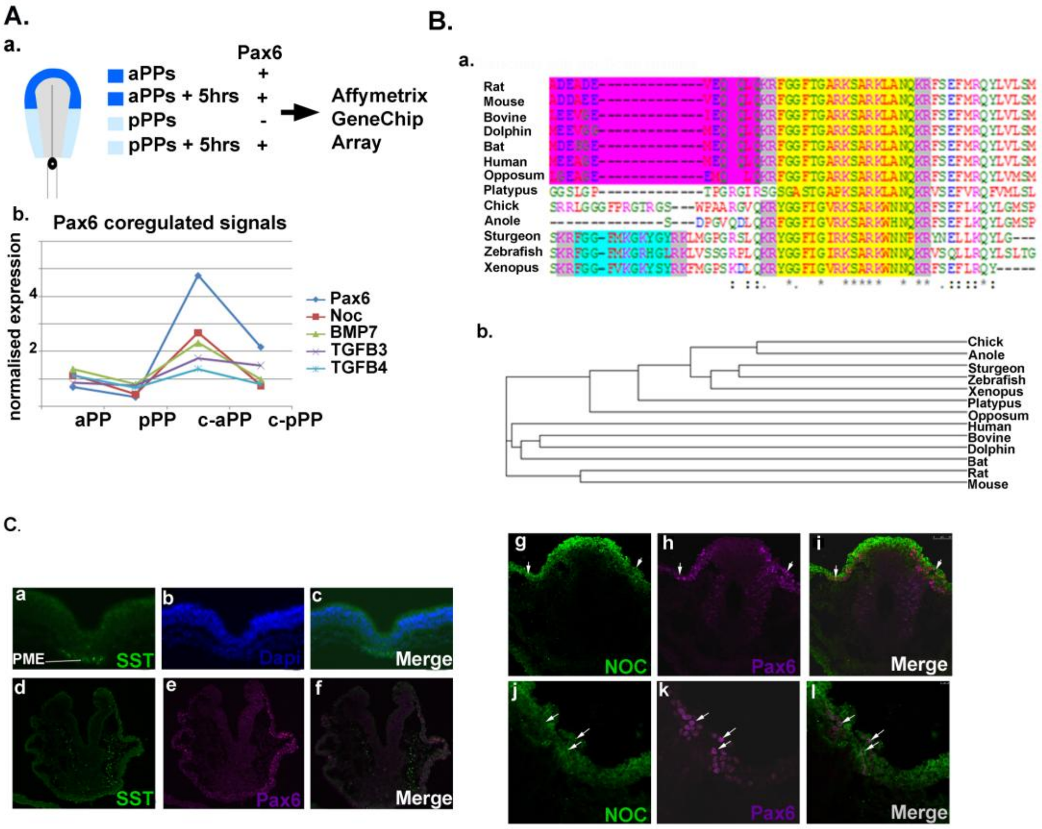

Figure S1, related to Figure 1. Nocicpetin and Somatostatin

A. Identification of new Pax6 regulators. a. Design of the screen: aPP and pPP explants from chick HH5/6 embryos were isolated and processed immediately or after 5hrs' culture. *Pax6* is absent in pPPs (Bailey et al., 2006). Transcriptomes were compared using Affymetrix genechip arrays. 1359 genes show significant changes of more than 2-fold (1-Way-ANOVA) in at least one cell population b. QT clustering and self-organizing maps identify 136 genes coregulated with *Pax6*, of which only four are signaling molecules including Noc.

B. Sequence analysis of nociceptin. Sequence alignment for the nociceptin propeptide**.** Grey: peptide cleavage sites; light blue: peptide F; yellow: nociceptin; purple: nocistatin. Color indicates different amino acids according to their physio-chemical properties: red, small hydrophobic including aromatic;

blue, acid; magenta, basic; green, hydroxyl+ amine + basic; grey, others. Symbols: two dots = change of amino acid with the same physio-chemical properties, * = conserved amino acid between all sequences. j. Cladogram showing the relation between different species based on the pronociceptin sequence. Non-mammalian vertebrates cluster together.

C. Localisation of somatostatin and nociceptin peptides. a-f. Double immunofluorescence on transverse sections of HH5 (a-c) and HH8 (d-f) embryos shows mesendodermal (pre-chordal mesendoderm: PME) expression of the SST peptide (a, c, d, f: SST, green), next to Pax6^{+} aPPs in the overlying ectoderm (e, f: Pax6 magenta). Nuclei are stained with DAPI (b, c: DAPI, blue). Note: Pax6 protein cannot be detected at HH5. e-h. Double immunofluorescence for nociceptin (g, I, j, l: Noc, green) and Pax6 (h, I, k, l: Pax6, magenta) reveals their localisation in ectodermal aPPs at HH8 (arrows); nociceptin is also present in neural folds, where Pax6 is absent. Nuclei are stained with DAPI (g, h: DAPI, blue). J-l are higher magnifications of the same sections shown in a-c; arrows: cells positive for both Pax6 and Noc.

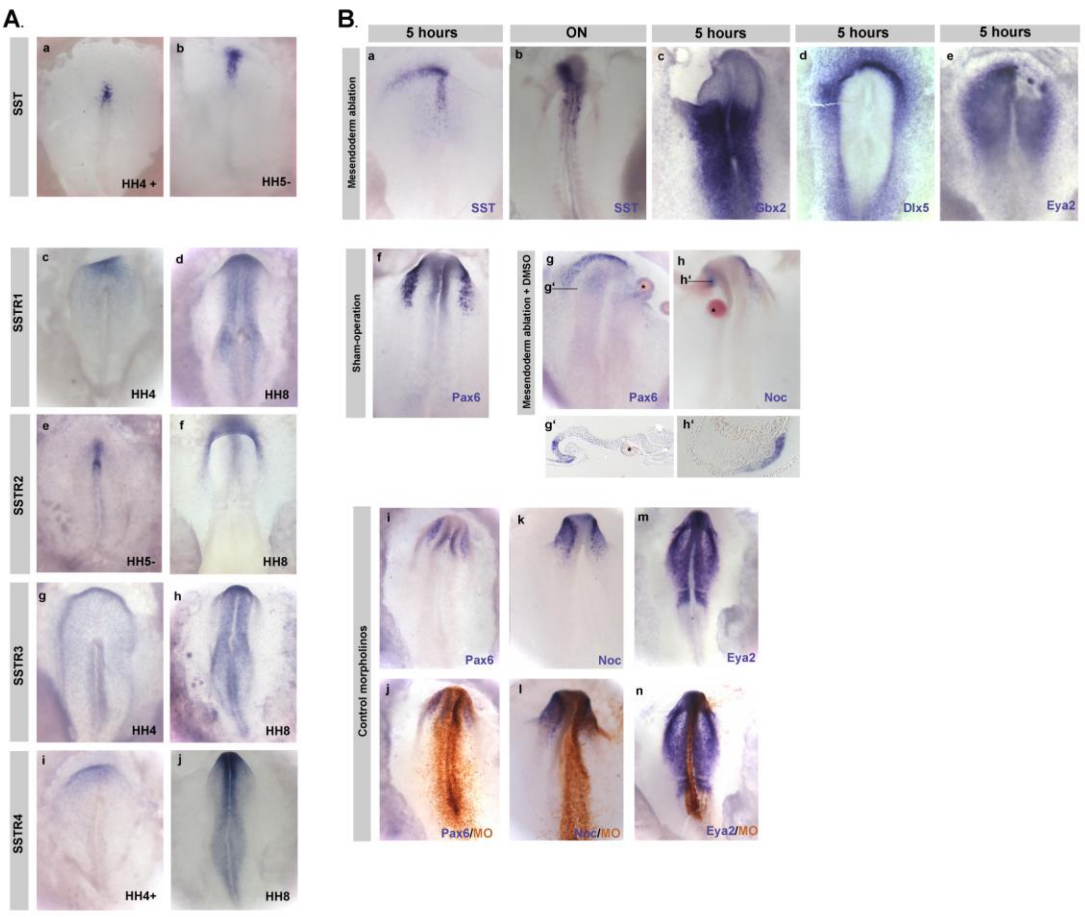

Figure S2, related to Figure 2. SST signaling.

A. Expression of SST and SSTRs. a, b: *SST* is expressed in the emerging head process. c, d: *SSTR1* is expressed in the anterior ectoderm at HH4 and in the neural plate and surrounding ectoderm at HH8. e, f: *SSTR2* is expressed in the node and emerging head process at HH5^{-} and in the endoderm at HH8. g, h: *SSTR3* is broadly expressed in the ectoderm at HH4^{-}, in the neural plate and surrounding ectoderm at HH8. i, j: *SSTR4* is expressed in the anterior ectoderm at HH4^{+}, in the neural plate and surrounding ectoderm at HH8.

B. Mesendoderm ablation and morpholino controls. a, b. Mesendoderm ablation removes SST source (right) after 5 hrs and overnight (ON). The expression of *Gbx2* (c) is not affected even after larger ablations; *Dlx5* (d)

does not change after mesendoderm ablation, while *Eya2* (e) is lost. f: Sham-operated embryo; *Pax6* expression. DMSO-coated beads do not rescue *Pax6* (g) or *Noc* (h) after mesendoderm removal. g' and h' show sections of the same embryos a the level indicated by the black line in g and h. Control morpholinos do not affect the expression of *Pax6* (i, j), *nociceptin* (k, l) or *Eya2* (m, n).

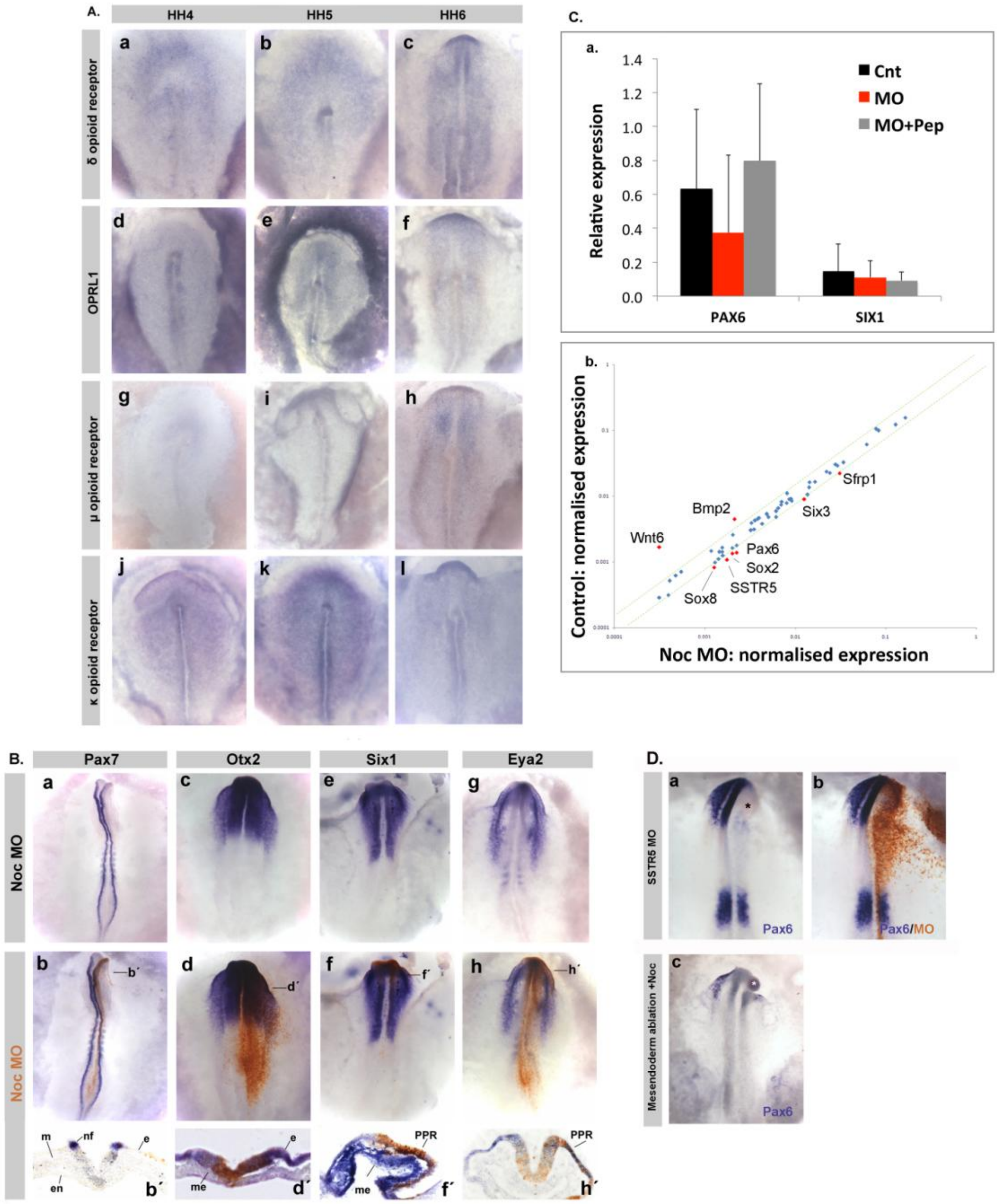

Figure S3, related to Figure 3. Nociceptin signaling.

A. Expression of nociceptin and opioid receptors. δ (a-c) and κ-opioid receptors (j-l) and OPRL1 are expressed broadly in the ectoderm from HH4 to HH6; μ-opioid receptor (g-h) is weakly expressed in the anterior neural plate.

B. Neural crest and pre-placodal markers are normal in Noc morphants. Nociceptin knockdown does not affect the neural crest marker *Pax7* (a-b’), the

anterior neural plate and placode marker *Otx2* (d-d') or the general PP markers *Six1* (e-f') and *Eya2* (g-h').

C. Noc knock-down leads to loss of *Pax6*. a: *Pax6* and *Six1* in explants cultured in control morpholinos (Cnt), Noc morpholinos (Noc) or Noc morpholinos and Noc peptide analyzed by qPCR. Diagram shows relative expression +/- SEM from 3 independent experiments; in each experiment 8-10 explants were pooled per condition. qPCR shows the same trend as in situ hybridization; however experiments vary, because on average only 65% of all explants upregulate *Pax6* and within each explant only a subset of cells is *Pax6*$^{+}$. b: NanoString N-counter analysis: Noc ATG morpholinos were electroporated unilaterally at HH4; at HH6 PP of electroporated and non-eletroporated side from the same embryo was collected and processed for NanoString analysis using a probe set containing PP markers Six1 and Eya2, aPP markers SSTR5, Pax6, Six3 and other genes irrelevant for the current analysis. Transcripts marked in red show more than 2-fold changes, while those between the dotted line do not change more than 2-fold. Transcripts above the diagonal are up-regulated, those below are downregulated in the presence of Noc morpholino.

D. Noc does not rescue mesoderm ablation or loss of SST signaling. a, b: HH4 chick embryos were electroporated with SSTR5-MO and received a graft of Noc-coated beads. Pax6 (blue) is absent on the electroporated side; b shows the same embryo as in a after visualization of MO (brown). c. mesendoderm was ablated unilaterally and replaced with a Noc-coated bead. Pax6 is absent on the ablated side.

Supplementary Experimental Procedures

Microarrays

aPP and pPP explants were isolated as described above; cells were lysed immediately or after 5 hours culture. Using 5ng of total RNA, labeled cell extracts were prepared and hybridized to Affymetrix Chick GeneChips (Chambers and Lumsden, 2008). Probe level values were derived from the raw data using the MAS5 algorithm (version 1.2; Affymetrix). Data were analyzed using the GeneSpring package (version 7.3.1; Agilent Technologies, Wokingham, Berkshire, UK). The suitability of the data sets for further analysis and the relationship between and within the biological replicates was determined using principle components analysis (PCA) and hierarchical clustering. Differential expression between the conditions under investigation was determined by a step-wise process: samples were first normalized to the 50th percentile across the whole expression dataset and then each gene was normalized to the median of its own expression across each cell type. Prior to statistical analysis, genes classed as being not expressed (absent in biological replicates) or not varying their expression above a twofold level in any of the cell types were removed from the analysis. From the remaining set genes, those whose expression levels differ significantly between each cell type was determined by one-way analysis of variance (ANOVA; $p = 0.05$). These genes were functionally classified using Gene Ontology (GO) criteria and were clustered into potentially Pax6 co-regulated groups using both Self-Organising Maps (SOMs) and Quality Threshold (QT [Pearson Correlation]) approaches. Data were deposited in Gene Expression Omnibus (GSE48116).

Analysis of DNA sequences

The nociceptin chick peptide was aligned using the multiple sequence alignment program ClustalW2 (http://www.ebi.ac.uk/Tools/msa/clustalw2/) with the respective proteins of other vertebrate organisms taken from Ensembl (http://www.ensembl.org/index.html). The program automatically generates a cladogram from the sequence alignment of the whole peptide.

Embryological manipulations

Fertilized hens' eggs from Winter Farms (UK), were incubated at 38ºC in a humid incubator until they reached the appropriate stages (Hamburger and Hamilton, 1951). Embryos were cultured in New culture (Stern and Ireland, 1981) or in modified Cornish pastry culture for long-term incubation (Nagai et al., 2011); mesendoderm ablations were performed at $HH5^-$. For rescue experiments, Ag1X2 beads coated with 100µM somatostatin peptide (TOCRIS bioscience), 100µM nociceptin peptide (YGGFIGVRKSARKWNNQ; Peptide Protein Research Ltd), or DMSO (control) were grafted into the ablated area; embryos were then cultured for 5 hours or overnight.

For inhibition of somatostatin and nociceptin signaling, stock solutions of cyclosomatostatin (Cyclo(7-aminoheptanoyl-Phe-D-Trp-Lys-Thr[Bzl]); CSST Sigma) and naloxone (Sigma) were prepared in DMSO (1mM); UFP101 (Sigma) was dissolved in H_2O (1mM). Embryos were preincubated for 1 hour in CSST, UFP101 and naloxone or all three (final concentration 1µM each), or in DMSO (0.1%) as control, and cultured in the presence of the same concentration of antagonist or DMSO for 5 to 60 hrs.

Morpholino electroporation

Knock down experiments were performed using morpholinos (Gene tools, LLC). Controls used standard control MOs (5'CCTCTTACCTCAGTTACAATTTATA3'); a MO targeting the transcription start site was designed for SSTR5 (5'GATCCATAACTCTTATTCCCCAGTT3'). For Nociceptin two MOs were used: one splice morpholino targeting the Exon2- intron3 junction and causing exon 2 deletion (5'CCGGCCATGTCGTACTCACTGGAGC3'); and one translation blocking MO (5'CAGAGCACGGCCCTCATGGTGACAA3'). MOs (1.6mM in 6% sucrose, 0.1% fast green; 0.2µg/µl pCAB DNA vector as carrier) were electroporated as described (Voiculescu et al., 2008) and embryos cultured until they had reached the desired stage.

Explant culture

pPP explants were obtained from HH5^{+}-6 embryos and cultured as described (Bailey et al., 2006). Collagen gels and culture media were supplemented as required: Trap 101 (1-[1-(Cyclooctylmethyl)-1,2,3,6-tet rahydro-5-(hydroxymethyl)-4-pyridinyl]-3-ethyl-1,3 -dihydro-2*H*-benzimidazol-2-one hydrochloride; Tocris bioscience; 500µM), 100 µM nociceptin peptide (YGGFIGVRKSARKWNNQ; Peptide Protein Research Ltd), 0.1 % DMSO, Endoporter (6 µl/ml; Gene Tools, LLC) in DMSO (0.1% DMSO final concentration), control MO (20 µM) and nociceptin splice MO (20 µM); FGF2 (R&D) 50µg/ml; SU5402 (Tocris) 10 µM. Experiments were repeated on at least three independent occasions; student t-test was performed to determine

significant differences. For co-culture with posterior head mesoderm, pPP and mesoderm were dissected separately and re-combined before culture.

Whole mount *in situ* hybridization and sectioning

Chick embryos and explants were processed for in situ hybridization (Streit et al., 1998) using DIG-labelled antisense probes for *Pax6* (Li et al., 1994), *somatostatin* (ChEST114e9 Geneservice^{TM}), *SSTR5* (ChEST679022 Geneservice^{TM}), *nociceptin* (ChEST781a9 Geneservice^{TM}), *Eya2* (Mishima and Tomarev, 1998), *Six1* (ChEST978K20 Geneservice^{TM}), *Otx2* (Bally-Cuif et al., 1995), *Ganf* (Knoetgen et al., 1999) and δ*crystallin* (Bailey et al., 2006). Zebrafish embryos were probed with *Pax6b*, *pnocb*, *Pitx3*, *Rx3*, *Emx1* and *Six3* antisense mRNA (Macdonald et al., 1994; Wilson et al., 1990). After incubation with anti-DIG antibodies (Roche), the colour was revealed using 4.5µl of nitroblue tetrazolium (50mg/ml) and 3.5µl 5-bromo-4-chloro-3-inolyl phosphate (50mg/ml) per 1.5ml of developing solution. For histological sections, chick embryos were embedded in paraffin and sectioned transversally at 15µm.

Immunocytochemistry

Immunocytochemistry in chick was performed after in situ hybridisation to reveal MO expressing cells using anti-FITC antibodies conjugated to peroxidise (anti-FITC-POD, sheep IgG; Roche; 1:500). For colour development embryos were incubated in 0.5mg/ml of 3, 3'-Diaminobenzidine (Sigma) in 100mM Tris, pH 7.4 and developed by adding H_2O_2 (Sigma) to a final concentration of 0.03%. For immunostaining on cryosections, embryos

were embedded in gelatin and sectioned at 10μm. The following antibodies were used: somatostatin (Abcam, 1:500), nociceptin (Abcam, 1:500) and Pax6 (Developmental Studies Hybridoma Bank, 1:50) and appropriate Alexa-coupled secondary antibodies (Invitrogen, 1:1000); nuclei were stained with 4',6-diamidino-2-phenylindole (DAPI).

Cloning of zebrafish pnocb and functional experiments

Zebrafish pnocb was cloned by RT-PCR using specific primers (5'-TGAAGTTCCTGCCTCATTCC-3'; 5'-GGTGCATGTCATGATGGAGA-3') from 48hpf embryo cDNA. Two MOs were designed targeting the translation start site (5'-GCAGGTCCAAAATGGAGTCTTCAT-3') and the intron1/exon2 junction (5'-CTCTCTGATGAAGGAGAGACAAGAA-3') of *pnocb* (Gene Tools. llC). 1-4 cell stage embryos were injected with either MO alone or both combined at a final concentration of 1.8 ng/embryo. Lower amounts of MO showed no effect. Identical phenotypes were observed in all cases. Embryos were grown at 28ºC until the desired stage. For somatostatin and nociceptin inhibition and rescue experiments using nociceptin, dechorionated embryos were incubated in antagonists or nociceptin peptide at the concentrations indicated in figure legends at 4-5 hpf until they had reached the appropriate stage.

RNA purification and qPCR from explants

HH6 pPP explants were treated with control morpholinos, Noc splice-blocking morpholinos or Noc splice-blocking morpholinos + Noc peptide as described in the methods section and cultured for 16-20 hours. 8-10 explants were

pooled for each condition in seven independent experiments. RNA was isolated using RNAaqueous Micro Kit (Ambion) and reverse transcribed using M-MLV reverse transcriptase (Promega). SYBR Green qPCR was carried out for Pax6 and Six1 and three housekeeping genes, GAPDH, HPTR and SDHA, for normalisation using a Rotor-Gene Q (Qiagen). Primers used were Pax6 forward 5’TCCTGATGTGTTTGCGAGAG3’, reverse 5’TGTTGCTGGCTTGTCTCC3’, Six1 forward 5’TACATAGCGGACGGTGAT3’, reverse 5’AATAACGACAGTGGAGAAGG3’; GAPDH forward 5’TCTCTGGCAAAGTCCAAGTG3’, reverse 5’TCACAAGTTTCCCGTTCTCAG3’, SDHA 5’GCTGTACATCTGCTCACACTAG3’, reverse 5’CTCTCCACGACATCCTTCTG3’, HPRT forward 5’AATTATGAAGGGCATGGGAGG3’, reverse 5’GACTTGTCACTGTTTCTGTTCAG3’. Relative expression levels were calculated using the Δ ΔC_t- method (Pfaffl, 2007). Experiments were performed in triplicates; t-test was performed to determine significance.

NanoString nCounter

For each experimental condition, 8-10 pPP explants were harvested and lysed in lysis buffer (Ambion). Total RNA from lysates was hybridized overnight at 65° with capture and reporter probes according to the nCounter Gene Expression Assay Manual. Following washing, target/probe complexes were eluted and immobilized for data collection in the nCounter Digital Analyser. Each experiment was repeated three times on independent occasions. Mean value +/- standard deviation for Pax6 and Noc was extracted

from the data normalised data; t-test was used to determine significant differences.

Embryos were electroporated with control or Noc splice-blocking morpholinos at $HH4^-$; at HH6 aPPs were dissected from the electroporated and non-elelctroporated side of each embryo. Tissue from individual embryos was lysed in lysis buffer and processed for NanoString nCounter.

References

Bailey, A.P., Bhattacharyya, S., Bronner-Fraser, M., and Streit, A. (2006). Lens specification is the ground state of all sensory placodes, from which FGF promotes olfactory identity. Dev Cell *11*, 505-517.

Bally-Cuif, L., Gulisano, M., Broccoli, V., and Boncinelli, E. (1995). c-otx2 is expressed in two different phases of gastrulation and is sensitive to retinoic acid treatment in chick embryo. Mech Dev *49*, 49-63.

Chambers, D., and Lumsden, A. (2008). Profiling gene transcription in the developing embryo: microarray analysis on gene chips. Methods Mol Biol *461*, 631-655.

Hamburger, V., and Hamilton, H.L. (1951). A series of normal stages in the development of the chick embryo. J Morph *88*, 49-92.

Knoetgen, H., Viebahn, C., and Kessel, M. (1999). Head induction in the chick by primitive endoderm of mammalian, but not avian origin. Development *126*, 815-825.

Li, H.S., Yang, J.M., Jacobson, R.D., Pasko, D., and Sundin, O. (1994). Pax-6 is first expressed in a region of ectoderm anterior to the early neural plate: implications for stepwise determination of the lens. Dev Biol *162*, 181-194.

Mishima, N., and Tomarev, S. (1998). Chicken Eyes absent 2 gene: isolation and expression pattern during development. Int J Dev Biol *42*, 1109-1115.

Macdonald, R., Xu, Q., Barth, K.A., Mikkola, I., Holder, N., Fjose, A., Krauss, S., and Wilson, S.W. (1994). Regulatory gene expression boundaries demarcate sites of neuronal differentiation in the embryonic zebrafish forebrain. Neuron *13*, 1039-1053.

Nagai, H., Lin, M.C., and Sheng, G. (2011). A modified cornish pasty method for ex ovo culture of the chick embryo. Genesis *49*, 46-52.

Pfaffl, M.W., 2007. Relative quantification. In: Real-time PCR, Dorak, M. T. (Ed.), Taylor & Francis, Oxford, UK. 63-80.

Streit, A., Lee, K.J., Woo, I., Roberts, C., Jessell, T.M., and Stern, C.D. (1998). Chordin regulates primitive streak development and the stability of induced neural cells, but is not sufficient for neural induction in the chick embryo. Development *125*, 507-519.

Wilson, S.W., Ross, L.S., Parrett, T., and Easter, S.S., Jr. (1990). The development of a simple scaffold of axon tracts in the brain of the embryonic zebrafish, Brachydanio rerio. Development *108*, 121-145.
